# Sketches of the Most Prevalent Diseases of India; Including a Treatise on Epidemic Cholera, &c.

**Published:** 1826-01

**Authors:** 


*Sketches of the most prevalent Diseases of India: comprising a Treatise on the Epidemic Cholera of the East; Statistical and Topographical Reports of the Diseases in the different Divisions of the Army under the Madras Presidency; embracing also the Annual Rate of Mortality, &c. of European Troops: and Practical Observations on the Effects of Calomel on the Alimentary Canal, and on the Diseases most prevalent in India.* By JAMES ANNESLEY, Esq. Madras Medical Establishment; lately in charge of the General Hospital, Madras; and Garrison Surgeon of Fort St. George. One volume, 8vo. pp. 464, with coloured Plates, price 18s. boards. Underwoods, October, 1825.

IT will not be denied that, during the last twenty or twenty-five years, many valuable additions to our pathological and therapeutical knowledge have emanated from our Indian practitioners. But it is not a little curious that, for several years previously to that period, the work of a London physician, expressly got up for the sake of acquiring wealth, and by one who never, it may be said, was out of the sound of Bow-bells, stood forth as the oracle to be consulted by all who embarked for, or returned from, that imperium in imperio, the Company's Possessions in the East. Every thing has its day—some a Summer's day, all sunshine—others, a short blink of light, and then perpetual darkness and oblivion! Hope, however, springs eternal, and books, like men, are immortal in their species, though transitory and destined to annihilation in their individual capacities.—Like men too, some books have an inherent tenacity of life, and run far and long on the stream of time, while others bring into the world with them such a principle of decay, as speedily saps the foundation of their existence, and consigns them to an early tomb. These reflexions apply, of course, only to things that *were*. We have no certain knowledge of any thing but the past—and, therefore, we are cautious in hazarding opinions respecting the future. Our humble occupation is that of a chronicler of the times—of the actual events passing around us, and rarely do we dare to anticipate what time alone can sanction or condemn. The PRESS is the midwife-general of the brain, and brings forth a numerous progeny, good and bad;—CRITICS stand forth in great numbers to baptise—but TIME alone can confirm!

In proportion as facts are more permanent than opinions, the work before us bids fair for its share of immortality. It is evidently the result of experience and careful observation, and we hope it will prove as serviceable to the author's brethren in the East, as it is creditable to himself on his native shores.

The work is divided into three parts, the first being in itself a treatise on the *late* (may it be so hoped) epidemic cholera of India, occupying no less than 250, out of 464 pages. This we conceive to be too large a proportion of occupation in a work purporting to be on "the most prevalent Diseases of India," and doubtless it will be so considered, after the authentic documents which have already issued from the three Presidencies respecting cholera—reports which unfortunately render it but too evident that the epidemic in its height was, in a majority of cases, little controlled by the powers of the healing art!

The second part of the work consists of topographical and statistical reports of the prevailing diseases in the different stations and divisions of the army under the Madras Presidency, occupying about 120 pages—while the third, or last part, contains about 90 pages of letter-press, on the effects of calomel on the mucous surface and secretions of the alimentary canal, and on the use of this remedy in disease, &c.

From this enumeration alone, it will be evident that the work is mis-titled. It should have been called "a Treatise on the Epidemic Cholera of India, with Statistical Returns, and Observations on the Use of Calomel." We make this remark merely for the purpose of shewing the connexion which ought to subsist between names and things—and to let the reader know what it is that he buys. We suspect that the title of the work was the suggestion of the book-*seller* rather than the author—the former being a personage who has an uncommon good turn for christening books, not by their real names, but by those which read well in the newspapers, the catalogues, and the shop windows. In all other respects, we need hardly say that the misnomer is of no consequence whatever.

In respect to the treatise on cholera, we confess that Mr. Annesley has more courage than we possess, in bringing out so large a work on an *epidemic* which is past, and which, according to his own opinion, never before occurred as such. If it never occurred before, we have reason to hope it may never again recur—or, at all events, that its visits may be at very widely distant intervals. But the main danger lies in publishing a new work on a disease which has been officially treated of—and that most minutely, by the Medical Boards of the three different Presidencies, as our readers well know—besides in numerous detached papers that have appeared in periodical works during the last four or five years. However highly we may estimate the talents of an individual, (and we have a high opinion of Mr. Annesley's abilities) still we cannot but be somewhat sceptical as to the power of an individual in adding much to reports that have been already most voluminous. In consonance, therefore, with the timidity which we confess, we shall not venture to go deep into the subject of the Indian cholera, in the present article; but merely notice the main features of the opinions and practices advanced or advocated in the work before us.

As we said before, Mr. A. does not think that there are any records or proofs of cholera, as an *epidemic*, having ravaged the Indian world before. He admits, however, that it has appeared *endemically* and *sporadically*, but then, of a less malignant nature.

Over the symptomatology of cholera we must pass entirely, with this remark, that our author seems to have paid more attention to the premonitory symptoms than any preceding writer. We were somewhat startled, however at finding that this epidemic cholera differs from all other epidemics "in the rapidity with which it runs its course—thus putting at defiance all human means of checking its progress." If this be the state of the case, why publish any more on the subject? the less that is said the better! But Mr. Annesley does not mean to draw so humiliating a picture of the healing art; though we much fear that, during the height of the epidemic, in some places at least, the above was, bonâ fide, the case.

Of the *pathognomonic* symptoms, "a burning sensation between the scrobiculus cordis and umbilicus," was the most constant—nay, our author asserts that he never saw an instance without it. But what is more curious, this was "precisely over that spot where the vermilion blush was invariably found on examination after death." This blush was situated in the small intestines, and exactly resembled the colour which they assume, when injected to shew the villi.

> "This symptom, therefore, I consider as particularly characteristic of the epidemic cholera; and this morbid appearance, which is related to it, I conceive to be the particular lesion which is uniformly to be met with on dissection of cases of the disease." 38.

A strong *diagnostic* mark was a black, thick, and ropy condition of the blood taken from a vein, or even an artery.

*Pathology of the Disease.* Twelve fatal cases with their *post-mortem* examinations, are minutely detailed, as the basis of our author's pathological conclusions. Many more dissections might have been added, but the appearances were so uniform, that he considered it unnecessary to relate them. These appearances are very remarkable, and we shall give them in the author's own words. Passing over the external appearances, and also the marks of congestion, with black blood in the head and thorax, we shall come at once to the abdomen, the apparent head-quarters of the disease.

"ABDOMEN.—Upon opening the abdomen, a peculiar offensive odour, as remarked by Mr. Jamieson, in his report of the Medical Board of the Bengal Presidency, respecting this disease was sometimes observed, particularly in those who died suddenly. The *stomach* generally contained more or less of a watery, muddy, and sometimes a grumous fluid. The colour of this fluid was various; sometimes it was colourless, at other times greenish, or passing to a yellow tint; and in some cases it was brown, approaching to black. The peritoneal surface of the organ seldom presented any other appearance than a greater congestion of the veins than was natural. The mucous surface was sometimes covered by a dark-coloured slimy mucus, and when this was removed, considerable congestion of the venous capillaries was observed. This congestion seemed to be chiefly seated in the submucous cellular membrane, and was occasionally so extensive in particular points, as to give the appearance of ecchymosis of this coat. The internal tunic was occasionally much corrugated, seemingly much thickened, and doughy to the touch, more especially when it was not much distended by fluid or flatus. The stomach was frequently flabby and relaxed, and its coats could be more easily penetrated by a harder body than usual. In those cases, in which some degree of re-action of the vital energies had taken place, the internal surface of this organ, particularly about the pylorus, presented a livelier colour, approaching to red, and was apparently thickened and contracted.

"The omentum was sometimes corrugated, or thrown to one side of the abdomen.

"The *small intestines* were, occasionally more than usually constricted in parts, frequently distended by flatus, and their veins generally engorged with black blood; externally, they presented a doughy thickened appearance, and their colour varied from a pale vermilion, through all the deeper shades, to a dark purplish hue; the former being chiefly remarkable on the peritoneal surface of the duodenum and jejunum, the latter in the ileum, about where it terminates in the cæcum. These shades of colour appeared to arise from the different degrees of congestion in the capillaries and veins in different parts of the canal, from the injection of the arterial capillaries, and from the colour of the blood which the vessels contained.

"When the small intestines were laid open, their coats seemed thickened, especially if the intestine was not distended, or if it was in any degree contracted; they were frequently flabby, and more easily torn than usual. The internal surface was generally found covered by a viscid, thick, and clay-coloured substance, which sometimes passed to a cream, or yellowish tint. This was particularly remarked in those who died after a sudden and short attack of the disease. When this matter was removed, the mucous coat itself was usually pale in the upper portion of the small intestines, and dark-coloured and congested in the lower part, particularly where the ileum is blue or purplish externally. When the disease was of longer continuance, and more particularly when some re-action of the powers of the system had taken place, this viscid appearance was detached to a greater or less extent, and was floating in the fluid contents of the small and large intestines; and the mucous coat then seemed more vascular, and the arterial capillaries appeared more injected, than in the former class of cases.

"The *large intestines* were frequently contracted, sometimes they were distended, and at others, they were both contracted and distended in different parts, in the same case. Congestion of the veins and venous capillaries was generally evident, especially of those seated in the cellular substance connecting the tunics. The external coat was generally dark-coloured, owing to the blackness of the blood in the congested vessels. The mucous surface was frequently very vascular; sometimes it presented a dark red colour, especially if the patient had lived for some time, and strong stimulants had been administered. These intestines never contained any fæces, and the fluids met with in them were generally similar to those found in the stomach and small intestines." 123.

"The *liver* was generally darker than natural, and loaded with black, thick blood. Sometimes this organ assumed a purplish, or dark blue colour; at other times it was mottled, enlarged, flabby, or pulpy, and easily torn.

"The *gall-bladder* was always distended by thick viscid bile, which was generally of a dark-green or black colour, in subjects who died before the appearance of bile in the excretions; and although the hepatic duct was large and permeable, the mouth of the common duct was generally constricted, and seldom permitted the bile to flow into the duodenum without considerable pressure made upon the gall-bladder. In those cases which terminated fatally after an illness of long duration, and in which some re-action of the vital energies and a flow of bile into the intestines, had taken place, the gall-bladder was generally empty, or contained but a small quantity of healthy bile; and the common duct, although not always free from some degree of constriction, was generally more permeable than in the former class of cases. In a few instances the gall-bladder was quite empty, relaxed, and flabby. In almost all the cases wherein bile was observed in the excretions, and the gall-bladder was found empty on dissection, and consequently, when it could be legitimately inferred that this secretion had passed into the intestines during the life of the patient, I remarked, that the viscid matter usually found lining the mucous surface of the small intestines, in the former description of cases, was detached to a greater or less extent, and was either floating in the fluid contents of the large intestines, or entirely removed, along with the matters which had been ejected from them.

" The *spleen* was generally enlarged, and engorged with black blood; and its texture was frequently soft. In some cases it fell to pieces whilst the examination of it and the adjoining parts was being performed, owing as much to an inordinate degree of distention, as to relaxation or softening of its texture. The colour of this viscus was uniformly darker than usual." 125.

" The *blood.*—The peculiar appearance of the blood, particularly excited my attention in the first case of the disease which came under my care. In every dissection which I performed, I uniformly found the venæ cavæ, the mesenteric veins, the veins in the vicinity of the heart, the vena portæ, the iliac and subclavian veins, and the sinuses of the brain, loaded by a thick, viscid, and black blood. The right cavities of the heart were generally distended with the same description of blood, and when any was found in the left cavities of this organ, it was similar in appearance to that lodged in the right. The lungs were always completely engorged with blood, of a pitchy or black appearance, and all the internal viscera presented a greater or less degree of congestion of blood, possessing nearly the same characters. The blood-vessels at the external surface of the body and in the extremities, were generally contracted and empty, or nearly so.

" That this condition of the circulating fluid was not consequent on death, although it might be more or less heightened thereby, is evident from the appearances which this fluid exhibited when taken away from a patient, even at an early period of the disease. During the subsequent stage, and more especially as the disease advanced to a fatal issue, the particular characters of the blood which have been now noticed were the most manifest, as may be seen in the details of the foregoing cases. That this state of the blood was the first material derangement consequent on the invasion of the efficient cause of the malady, I shall not contend; but that it was one of the earliest links in the chain of effects consequent to that cause, and that it afterwards tended, by a necessary and evident process, to heighten and to perpetuate the derangement whence itself sprung, I have not the least doubt. That the nervous influence, in some manner or other, received the first impression of the morbid cause, and afterwards gave rise to this condition of the circulating fluid, may be inferred, if we be permitted to conceive that a diminished function of the lungs, liver, and other excerning viscera was co-existent, or nearly so, with that primary change; and consequently, that the blood did not undergo an elimination of its effete and noxious constituents, to an extent requisite to the performance of the organic actions and the continuance of life." 127.

The appearances on dissection, both as respects the solids and fluids, were precisely the same in the natives of the country, as in Europeans—the only difference being the more speedy fatality in the former, and the congestion being in a greater degree.*

*Etiology.* Our author first adverts to the *proximate* cause, which, as may be anticipated from the foregoing statements, he conceives to consist in "a singular and sudden change in the circulating fluid," as evinced by the extreme venous congestion, and the black viscid state of the blood. This change, he thinks, and not unreasonably, must be, "the effect of some uncommon influence over the vital powers." But what is this influence—and how does it effect these changes in the blood? That is the question; but who can answer it? Is it answered by our author in the following passage? We fear it is not.

"From these facts and considerations, therefore, I am led to conclude, that either the absence of electricity from the human body, or some important change in its electrical state, arising, perhaps, from exposure to a negative electrical atmosphere, may be the cause of the dreadful and destructive epidemic which has recently ravaged the East; and that the vicissitudes of the seasons preceding this formidable visitation may support this opinion. If, therefore, this view of the subject be correct, we may readily account for the sudden attacks of the disease, the changes in the temperature and sensibility of the body, and in the fluids, which changes seem chiefly to characterise it, and for the manner in which it has been limited to some districts, extended to others, and has successively ravaged all." 139.

We are ready to admit that this conjecture (for assuredly it is no more) is just as feasible as any that have been offered—and much more so than some that have been emitted by the spectators of this frightful epidemic. It is curious that Mr. Annesley observed a black and sizy state of the blood, "in almost every case where he had occasion to perform venesection, whether in cholera, dysentery, fever, hepatitis, or rheumatism," during the last four or five years.

No probable efficient cause, however, for this change in the fluids, could be discovered in the sensible conditions of the weather or the seasons. The disease prevailed in hot, cold,

---

* We may here remark that *every appearance* recorded by Mr. Annesley is also recorded by Mr. Scott, (*see Med. Chir. Rev. No.* 5, *p.* 131, 132) with this difference, that so far from the *post-mortem* phenomena being uniform, they exhibited "such a variety of appearances that no particular light was thrown on the disease by them."—Here then is a discrepancy in matters of fact which cannot fail to operate seriously against the exclusive pathology of Mr. Annesley.

wet, and dry weather—in low and in elevated situations—and, in short, "under all cognizable circumstances."—"It must have depended, therefore, upon the influence of some quality in the atmosphere which has been overlooked, or which was, perhaps, beyond the reach of human observation."

Mr. Annesley observes that it is a well-known fact that "in the very centre of extensive districts ravaged by epidemic cholera, there are certain *narrow stripes or patches* of country into which the disease has never penetrated, though all around was one scene of desolation.*" This circumstance, he thinks, "militates most conclusively against any idea as to its being a contagious disease." We confess that we do not see the necessity of this conclusion—and we venture to predict that Sir Gilbert Blane will adduce this very circumstance in favour of contagion. At all events, it surely militates at least as much against any general atmospheric influence. It comes much more to the support of the opinion which we ourselves have always maintained, that epidemic as well as endemic diseases depend on emanations from the earth. This conclusion, we think, is virtually embraced by Mr. Annesley himself in the following passage.

"This limitation of the disease to places where there existed no natural obstacles to its extension, militates most conclusively against any idea as to its being a contagious disease, and seems to point to the existence of some difference in the quality of the atmosphere; and I have little difficulty in believing that difference to have been chiefly in its electrical state; *which state may also have had an intimate relation with the exhalations proceeding from the soil of the places where the disease was predominant.*" 146.

The general character, he observes, of the invading symptoms favours this opinion. These symptoms more or less suddenly supervened, and the vital functions seemed overwhelmed as if some poison had been taken into the stomach, or infused into the blood. It is a curious fact, Mr. A. further remarks, that the appearances after death in cholera were not at all unlike those which present themselves in persons who have died from the effects of narcotic poison, as the poison of the cobra de capella, &c.

Our author, therefore, regards epidemic cholera as "essentially an affection of the nervous system," and considers the diminution of the nervous power to be the proximate effect of the efficient cause of the disease—"that cause being the *electrical condition of the air*, arising from, or accompanied by

* For instance, the hill-forts in Kandeesh.

*terrestrial exhalations* of a kind unfavourable to life." It is hardly necessary to say that both of these are pure conjectures —and that neither of them has any title to originality—the first proposition having long ago been maintained by Mr. Orton, and the latter by ourselves.

Treatment. Viewing the disease as one of apparent debility, whilst *oppression* of the vital powers is the real cause—our author's indications of cure are—" to remove oppression from the venous system, and restore the balance of the circulation." Our readers need not be told that these are the very indications which we ourselves laid down thirteen years ago. " Bleeding, therefore, where it can be effected, should never be lost sight of." This measure was first suggested by Dr. Johnson, and has since been much acted on by various practitioners in India, and often with good effect. It can only be put in force in the early stage of the disease, and before the circulation leaves the wrist.

It is supposed by some, and not denied by Mr. Annesley, that death has sometimes been accelerated by venesection ; but in such cases he thinks death would have been inevitable, whatever were the means employed.

" We have instances, however, wherein blood, drawn even in the advanced stage of this disease, has continued to flow till the balance of circulation was restored, and the patient recovered.

" In these instances the blood was at first thick, black, and came away in drops ; at length it became thinner, and flowed with more ease, till the colour changed to a bright red. This is the change which should always be looked for, and whether it take place after the abstraction of one ounce or thirty, is of no consequence ; this change must supervene before the patient can be considered safe. Under all circumstances, therefore, I think we should never forego a trial of the lancet." —169.

Although Mr. Annesley recommends bleeding at all times, he does not mean to deny that many successful cases have occurred where it has not been used at all—" nor does he answer for its universal success." But he ventures to assert that, if it can be accomplished in the early stage of the disease, and before the circulation has ceased at the wrist, " in nine cases out of ten it will prove successful, especially if the colour of the blood change from black to red, if the pulse get up, and the spasms be relieved." 170.

Our author uses, as auxiliaries, camphor, ammonia, ether, rubefacients, nitric acid blisters, the warm vapour bath.

"The following is the way in which this disease has usually been treated under my direction :—

"A patient is admitted into the hospital, I shall say at noon, with all the symptoms of cholera; a vein is immediately opened, and one scruple of calomel and two grains of opium are given in the form of a pill, and washed down with the camphor draught. The body and extremities are well rubbed with dry flannels made warm, and bottles filled with hot water are applied to the feet and hands; but if the spasms are severe, spirits of turpentine are used as an embrocation. In an hour we generally perceive the effects of these remedies, and whether the disease be in any degree arrested, or be proceeding in its progress. If the former, nothing more is to be done till evening, when the calomel pill may be repeated, and an enema exhibited. The following morning the bowels should be again fully evacuated, and then the patient may be considered safe.

"When blood, however, cannot be drawn from the arm, and the spasms continue; when severe pain and burning heat are felt at the umbilicus and scrobiculus cordis, and are distressing; when the skin is cold, and deluged with cold, clammy dew; and when there are oppression in the chest and difficulty of breathing—excessive pain and confusion about the head, with great intolerance of light—no pulse, or a pulse scarcely to be felt, and a cadaverous smell from the body, twenty or thirty leeches should be applied immediately to the umbilicus and scrobiculus cordis; the calomel pill should be repeated, and the turpentine embrocations continued. Leeches ought likewise to be applied to the temples and base of the skull.

"When the leeches bleed freely, the application of them is always attended with decided advantage, and they should be allowed to remain till they have fulfilled their duty; after which, a large blister or sinapism should be applied over the whole abdomen. Sometimes the leeches fasten, but do not draw blood. In this case they should be removed immediately, and the sinapism or blister applied in their place. When the bowels are very irritable, and constantly discharging a watery fluid, small anodyne enemas, with camphor, may be given; and the drogue amère, a nostrum used by the Jesuits, will be then found very useful in assisting the operation of calomel, which latter should always be repeated every two hours, till three or four scruples have been taken.

"Whenever we fail in checking the disease at first, we have no resource but to treat urgent symptoms, and they must always be met with decision as they occur. The patient ought never to be left a moment without an attendant who is capable of acting according to circumstances, and who may take advantage of every change." 180.

As, on dissection, the bowels have been found diseased, and the intestines lined with a thick, viscid matter, like old cream cheese, which glues them together and obstructs the passage, this matter ought in every case to be removed, if possible. Purgatives did not seem to effect this object in our author's practice.

"Calomel, in scruple doses, I have always found most useful in removing this particular matter. Sometimes I have combined it with aloes, and continued it every night and morning, till the dejections became of a blackish grey colour, substantial and tenacious. The purging draught and the enema may then be had recourse to, with the best effects.*" 188.

Mr. Annesley next details numerous successful cases illustrative of the mode of treatment above described, both in his own practice and that of others, making many judicious reflections and remarks on the nature of the disease and the best mode of fortifying and preserving the constitution against its attacks. These will be perused with great interest and advantage by all those who visit our Indian territories, and to them we strongly recommend the work. The other subjects treated of in this volume, will find a place in another part of our work.

---

### VIII.

"* Three, four, and even five scruples of calomel were usually taken, before this effect was produced; and the black, grey colour seemed always indicative of the action of calomel, being precisely the colour which is produced by calomel combined with ammonia."

